# Development and Future Prospects of Bamboo Gene Science

**DOI:** 10.3390/ijms26157259

**Published:** 2025-07-27

**Authors:** Xiaolin Di, Xiaoming Zou, Qingnan Wang, Huayu Sun

**Affiliations:** 1 Key Laboratory of National Forestry and Grassland Administration on Bamboo & Rattan Science and Technology, Beijing 100102, China; dixiaolin@icbr.ac.cn (X.D.); zouxiaoming@icbr.ac.cn (X.Z.); wqn0716@gmail.com (Q.W.); 2 Institute of Gene Science and Industrialization for Bamboo and Rattan Resources, International Centre for Bamboo and Rattan, Beijing 100102, China; 3 National Academy of Innovation for Bamboo as a Substitute for Plastic, Beijing 100102, China

**Keywords:** bamboo, gene science, genome sequencing, gene editing, bamboo as a substitute for plastic

## Abstract

Bamboo gene science has witnessed significant advancements over the past two decades, driven by breakthroughs in gene cloning, marker-assisted breeding, sequencing, gene transformation, and gene editing technologies. These developments have not only enhanced our understanding of bamboo’s genetic diversity and adaptability but also provided critical tools for its genetic improvement. Compared to other crops, bamboo faces unique challenges, including its long vegetative growth cycle, environmental dependency, and limited genetic transformation efficiency. Then, the launch of China’s “Bamboo as a Substitute for Plastic” initiative in 2022, supported by the International Bamboo and Rattan Organization, has opened new opportunities for bamboo gene science as well as for bamboo production systems. This policy framework has spurred research into bamboo genetic regulation, fiber-oriented recombination, and green separation technologies, aiming to develop sustainable alternatives to plastic. Future research directions include overcoming bamboo’s environmental limitations, improving genetic transformation efficiency, and deciphering the mechanisms behind its flowering. By addressing these challenges, bamboo genetic science can enhance its economic and ecological value, contributing to global sustainability goals and the “dual-carbon” strategy.

## 1. Introduction

Bamboo, a globally significant forest resource, offers extensive ecological, economic, and social benefits [1]. With over 1400 species distributed worldwide [2], bamboo forests cover approximately 35 million hectares. China, home to the world’s richest bamboo resources, accounts for over 7.56 million hectares, providing a fertile ground for germplasm innovation and the development of new bamboo varieties [1,3,4]. The bamboo culm, the most valuable part of the plant, develops from bamboo shoots and completes its height (10–20 m) growth within 1.5 months. Bamboo fiber production begins at 1–2 years, with the material reaching its optimal state in 3–5 years [5]. Given its rapid growth and versatile applications, bamboo is increasingly recognized as a sustainable resource in global forestry, environmental ecology, and sustainable development.

Bamboo exhibits superior material properties, characterized by remarkable toughness and mechanical strength. When bamboo walls undergo delignification and thermal compression, they transform into high-density bamboo sheets with exceptional tensile strength, surpassing that of various natural wood materials, engineered steels, and high-strength metal alloys. This advanced material is often referred to as “bamboo steel” [1,6,7]. Furthermore, the diverse morphologies of bamboo culms, including variations in internode length, diameter, wall thickness, node characteristics, and color, are closely linked to their utilization. Species like Moso bamboo and Giant Dragon bamboo (*Dendrocalamus sinicus*) are ideal for timber, while others like Dwarf bamboo (*D. strictus*) and *Neosinocalamus affinis* are suited for papermaking. Unique varieties such as the Buddha Belly bamboo (*Bambusa ventricosa*) and purple bamboo (*P. nigra*) are prized for ornamental purposes. Additionally, bamboo species with thickened walls or solid culms serve as important germplasm resources, maintaining genetic diversity and offering broad application prospects globally. Understanding the genetic basis of these traits is crucial for future applications in ornamental, timber, and plastic-substitute bamboo varieties.

Data is sourced from the PubMed database (Search conducted on 26 June 2025, using keywords related to molecular markers, gene cloning, sequencing technology, and gene editing and heterologous expression).

**Figure 1:**
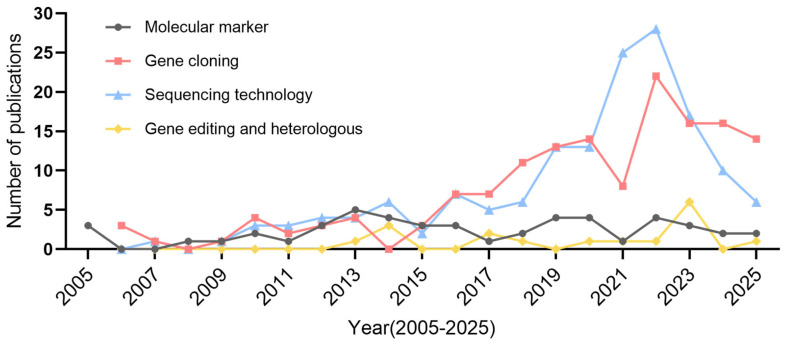
Trends in publication numbers of bamboo gene science research from 2005 to 2025.

Over the past two decades, bamboo gene science research has evolved from nothing to significant progress in areas such as molecular markers development, gene cloning, sequencing technology, and gene editing and heterologous expression, particularly with a surge in published papers in recent years (Figure 1). This indicates substantial development in bamboo gene science. Despite its ecological and economic significance, the progress of bamboo gene science has been significantly impeded by its unique biological characteristics [8]. Traits enhanced through gene overexpression frequently manifest only in the T0 generation and vanish in subsequent generations, as the degradation of the cytosolic mRNA of exogenous DNA fragments introduced into plants leads to gene silencing [9]. This implies that genetic improvements in bamboo via transgenic overexpression may not yield sustainable results, necessitating reliance on endogenous gene editing techniques. However, this approach introduces another challenge: the difficulty of eliminating foreign gene fragments through trait segregation in gene editing. These issues are not exclusive to bamboo breeding but are also encountered in other crops. Bamboo, however, presents unique breeding challenges. Firstly, its extended vegetative growth phase, which can last decades, combined with an unpredictable flowering cycle ranging from 3 to 150 years [10,11,12], renders traditional breeding methods inefficient. In the era of molecular breeding [8], bamboo was anticipated to achieve parity with other crops, yet it faced substantial obstacles. Secondly, low efficiency in genetic transformation and regeneration has long hindered genetic engineering improvements [13]. Recent advancements in establishing genetic transformation and gene editing systems in Moso bamboo and Ma bamboo (*D. latiflorus* Munro) have facilitated targeted genetic enhancements based on gene function: the agrobacterium-mediated transformation efficiency is approximately 5% in Moso bamboo and 0–4% in Ma bamboo, and there is still significant room for improvement [13,14,15,16,17]. One of the main reasons for the low efficiency of genetic transformation in bamboo is the difficulty in obtaining callus in an optimal state. Currently, only a few laboratories with specialized expertise have successfully achieved this state, as reported in published studies. When the callus does not reach this optimal condition, the transformation efficiency drops significantly. Another major bottleneck is the low efficiency and prolonged time required for shoot regeneration (dedifferentiation) [8]. Additionally, the time required for bamboo to grow from seeds or callus into usable timber is extensive. For example, Moso bamboo seeds planted in 2004 in Taiping, Anhui province, China, showed increased culm diameter over years of sprouting, but it took 20 years for the culms to reach a diameter of 9–10 cm, comparable to wild asexually propagated bamboo. This highlights that genetically transformed bamboo through tissue culture requires two decades to manifest the effects of genetic improvement. These aforementioned challenges, along with the need to consider the effects of climatic adaptation between different species and the importance of zoning studies to ensure bamboo plant materials express the best development conditions, collectively constrain the advancement of bamboo gene science.

In recent years, advancements in high-throughput sequencing technology and the promotion of “Bamboo as a Substitute for Plastic” policies have opened new avenues for bamboo gene science [17]. These developments have not only enhanced our understanding of bamboo’s genetic diversity and adaptability but also provided critical tools for its genetic improvement. This paper aims to review the development of bamboo genetic science, analyze its unique challenges and opportunities compared to other crops, and explore future research directions to enhance its economic and ecological value.

## 2. Development of Bamboo Gene Science

**Figure 2:**
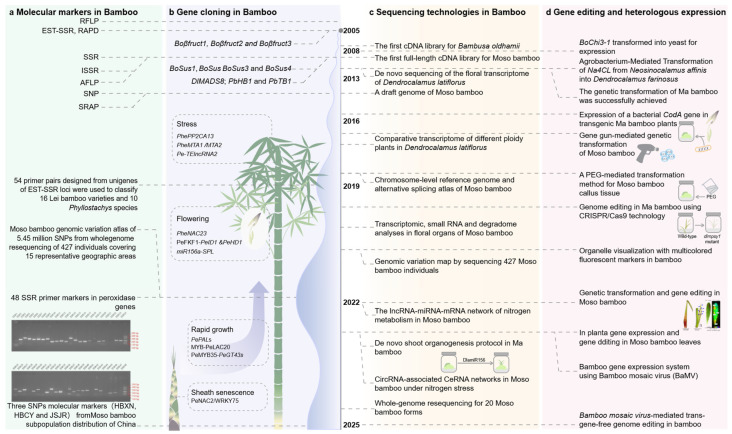
Timeline of bamboo gene science development. (**a**) Molecular markers in Bamboo [18]. (**b**) Gene cloning in Bamboo. (**c**) Sequencing technologies in Bamboo. (**d**) Gene editing and heterologous expression [19].

The field of bamboo gene science has witnessed remarkable progress, driven by cutting-edge technologies and innovative research approaches. This section delves into the key areas of development, highlighting how molecular tools and genetic techniques are revolutionizing our understanding of bamboo biology (Figure 2).

### 2.1. Application of Molecular Markers in Bamboo Classification

Traditional plant classification primarily relies on reproductive characteristics. However, most bamboo species flower only once in their lifetime and die shortly thereafter, with flowering cycles spanning over several decades [20,21]. This rarity of flowering events makes bamboo flower specimens exceptionally scarce. Consequently, vegetative features—such as rhizomes, branch types and numbers, culms, culm-sheaths, and leaves—have become essential for bamboo classification. Nevertheless, morphological variations in culms, leaves, or rhizomes during different developmental stages or under diverse environmental conditions often lead to taxonomic disputes, severely limiting the reliability of floral characteristics. Therefore, a detailed analysis of the environmental conditions that influence morphological variables is essential for a deeper understanding of the factors modulated by climate and for assessing their implications from the perspective of climate change. This complexity in bamboo classification highlights the challenges of relying on morphological traits. In contrast, the use of genetic information as molecular markers offers a highly effective and convenient alternative, revolutionizing the approach to bamboo taxonomy.

The use of genetic information as molecular markers presents a promising opportunity for bamboo classification (Figure 2a). Early applications of bamboo DNA focused on molecular markers for classification and evolutionary studies, even without specific sequence information. In 1994, nuclear restriction fragment length polymorphisms (RFLPs) were employed to analyze 20 species of the genus *Phyllostachys*, generating genetic distances and examining intra- and inter-species variation [20]. Subsequently, Gielis et al. [22] emphasized the potential of molecular markers for genotype identification (“fingerprinting”), genetic mapping, and precise biosystematic and evolutionary studies. Advances in molecular techniques introduced methods such as RAPD, ISSR, SSR, AFLP, SRAP, and EST-SSR for bamboo classification and evolutionary research [23,24,25,26,27,28]. However, the limited availability of sequence data restricted the identification of polymorphisms, hindering comprehensive analyses.

In the post-genomic era, the availability of extensive genomic data and the emergence of genome-wide SSR, EST-SSR, and SNP markers have significantly improved the accuracy of genetic DNA tagging [29,30,31,32,33]. The development of high-throughput sequencing technologies, such as illumina sequencing, Oxford nanopore technology and single-cell RNA sequencing, has addressed the challenges posed by bamboo’s long and irregular flowering cycles, making molecular markers invaluable for assisting bamboo taxonomy and advancing genomic studies in bamboo and related species.

### 2.2. Application of Gene Cloning and Functional Analysis in Bamboo

Bamboo exhibits complex chromosomal ploidy, encompassing four major lineages with a base chromosome number of 12 and varying ploidy levels. Herbaceous bamboos are diploid, while temperate woody bamboos and neotropical woody bamboos are either allotetraploid or allohexaploid. Palaeotropical woody bamboos are hexaploids [34,35]. Chromosomes, as carriers of genetic material, play a pivotal role in determining phenotypic traits, and variations in their number can lead to significant changes in genetic characteristics. Therefore, gene cloning and functional analysis in bamboo are crucial for elucidating its unique traits and advancing genetic improvement efforts.

The screening, cloning, and functional characterization of genes are critical steps in understanding the molecular mechanisms underlying various biological phenotypes. These processes are essential prerequisites for reverse genetics, which aims to identify and validate gene functions. Prior to the establishment of cDNA libraries and comprehensive genomic information, researchers primarily relied on homologous cloning—a time-consuming and labor-intensive method that utilized conserved sequences from related species, such as rice or other plants, to identify and clone target genes (Figure 2b). For instance, early studies employed homologous cloning to isolate genes like the Teosinte branched1 gene (*TB1*) from *P. violascens* [36]. Transcriptional analysis revealed its expression in shoots, leaves, and flowers, with in situ hybridization showing higher expression in lateral buds, indicating its role in shoot branching. Similarly, conserved sequences from Poaceae species were used to clone genes such as the light-harvesting chlorophyll a/b-binding protein gene (*cabBO1*), *PIF*-like transposable elements, antifungal class III chitinase gene, and cellulose biosynthesis genes (*PeCesA1*, *PeCesA2*, etc.) in bamboo [37,38,39,40,41]. These studies highlight the importance of gene cloning and functional analysis in understanding bamboo’s genetic architecture and its potential applications in genetic enhancement.

The post-genomic era has revolutionized gene functional analysis by providing easier access to genetic sequences and accelerating reverse genetics. This transformation has been particularly impactful in bamboo research, where significant advancements have been made in understanding non-biological stress response, flowering, rapid shoot growth, and sheath senescence [42,43,44,45,46,47,48] (Figure 2a). A key milestone in this progress was the establishment of the first cDNA library for green bamboo (*B. oldhamii*) in 2006 and the first full-length cDNA library for Moso bamboo in 2010 [49,50] These libraries eliminated the reliance on homologous cloning, enabling researchers to design primers directly from cDNA sequences and efficiently amplify target genes using techniques such as RT-PCR and RACE.

For instance, the cloning of *Boβfruct1*, *Boβfruct2*, and *Boβfruct3* encoding cell wall and vacuolar invertases, as well as the sucrose synthase genes (*BoSus1*, *BoSus2*, *BoSus3*, and *BoSus4*) in green bamboo shoots was significantly streamlined using cDNA libraries [49,51]. Further studies identified *Boβfruct1*, *Boβfruct2*, and *Boβfruct3* encoding cell wall and vacuolar invertases in green bamboo shoots. Transcriptional profiling revealed the growth-dependent expression of *Boβfruct1* and *Boβfruct2* in shoot base regions, highlighting their roles in sucrose unloading and shoot growth. *Boβfruct3* was predominantly expressed in regions of cell differentiation and expansion, suggesting functions in osmoregulation and cell enlargement, which are critical for sustaining the rapid growth of bamboo shoots [51].

Similarly, the isolation of *DlMADS8* from sweet bamboo (*D. latiflorus*) and its functional characterization in *Arabidopsis thaliana* demonstrated the utility of cDNA libraries in studying gene function. Transgenic plants exhibited curled leaves, early flowering, and novel inflorescence phenotypes, indicating DlMADS8’s role in floral meristem determinacy and flowering time regulation [52].

The advent of genome DNA and cDNA libraries not only accelerated gene cloning efforts but also enabled comprehensive transcriptional profiling and functional studies. For instance, Peng et al. [53] used a rice gene chip to detect gene expression in rhizome buds of *P. praecox* and isolated a novel *REVOLUTA*-like (*REV-like*) homeobox gene (*PpHB1*). Its expression in lateral bud tips and developing procambium suggested its involvement in rhizome bud formation and procambial development [53]. These findings underscored the role of cDNA libraries in uncovering gene regulatory networks.

In summary, the development of genome DNA and cDNA libraries has revolutionized bamboo gene cloning by eliminating the need for homologous cloning and enabling researchers to isolate and study genes more efficiently. This advancement has significantly contributed to our understanding of bamboo biology and its applications in sustainable agriculture and biotechnology.

### 2.3. Widespread Application of Sequencing Technologies in Bamboo

The rapid development of sequencing technologies in recent years, from single DNA fragment sequencing to whole-genome sequencing, and further to transcriptomics and single-cell omics, has significantly advanced the throughput and technical capabilities of bamboo gene science (Figure 2c). These technological breakthroughs have provided new perspectives for understanding the gene regulatory networks and metabolic pathways in bamboo.

The first full-length cDNA library for Moso bamboo was released in 2010 [50]. In 2013, a draft genome of Moso bamboo was assembled [54], which was later refined to the chromosome level [55]. Subsequently, Zhao et al. [30] constructed a comprehensive genomic variation map by sequencing 427 Moso bamboo individuals. These advancements in sequencing technologies have revolutionized bamboo gene science, providing unprecedented insights into its genome architecture, evolutionary history, and functional genomics.

With the decreasing cost of sequencing, especially transcriptomics, its application in bamboo research has expanded significantly. In 2010, five cDNA libraries for Moso bamboo were constructed from shoots just breaking out from the ground, shoots reaching a height of ~40 cm, young leaves, and shoots and roots removed from germinating seeds, and submitted to GenBank [50]. These libraries greatly facilitated gene cloning, expression analysis, and functional characterization. Then, Zhang et al. [14] performed de novo sequencing of the floral transcriptome of *D. latiflorus*, providing a valuable resource for studying floral transition and development in bamboo. Peng et al. [56] conducted transcriptome sequencing of Moso bamboo shoots at six different heights (10, 50, 100, 300, 600, and 900 cm) and culms after leaf expansion. Due to the importance of bamboo shoot development and the affordability of transcriptomics, multiple transcriptomes of Moso bamboo shoots have been published, reflecting diverse sampling strategies and research focuses [8,57].

Beyond conventional transcriptomics, studies have also explored degradomics, miRNAs, circular RNAs (circRNAs), and long non-coding RNAs (lncRNAs). Cheng et al. [58] used transcriptomic, small RNA, and degradome analyses to identify key miRNAs and their targets in the floral organs of Moso bamboo, shedding light on the molecular networks underlying floral development. Wang et al. [59] established a de novo shoot organogenesis protocol in Ma bamboo and revealed transcriptomic dynamics during regeneration, highlighting the potential roles of Ma bamboo miRNAs (DlamiRNAs) in this process. Yuan et al. [60] constructed a regulatory network of nitrogen metabolism, including 17 metabolic pathway genes, 15 transcription factors (TFs), 4 miRNAs, and 10 lncRNAs, providing new insights into the regulation of nitrogen metabolism in bamboo and facilitating genetic improvements for adapting to fluctuating nitrogen environments. Zhu et al. [61] identified 536 circRNA-parent genes unevenly distributed across 24 scaffolds and associated with root growth and development. Among these, 52 differentially expressed circRNAs (DECs) were found to act as miRNA sponges, participating in organ nitrogen compound biosynthesis and the metabolic process regulation of nitrogen, amino acid, and energy in Moso bamboo [61].

The release of high-throughput omics data for bamboo has provided critical resources for understanding its genetic diversity and adaptability to stressors such as low temperature, intense light, and drought, as well as the mechanisms underlying its rapid growth and flowering. These advancements pave the way for further exploration and the genetic improvement of bamboo species.

### 2.4. Application of Transgenic Overexpression and Gene Editing in Bamboo

The application of heterologous expression and gene editing technologies, such as CRISPR-Cas9, has significantly advanced the study of gene function in bamboo. These technologies not only elucidate the roles of genes in biological processes but also enable precise regulation of gene expression to modulate plant phenotypes, offering powerful tools for bamboo genetic breeding (Figure 2d).

Early approaches: heterologous expression in model organisms. Before the development of bamboo-specific transformation systems, gene function validation in bamboo relied on in vitro enzymatic assays in Escherichia coli or heterologous expression in model plants like rice and *Arabidopsis*. For instance, Kuo et al. [39] demonstrated the antifungal activity of the green bamboo class III chitinase gene against Scolecobasidium longiphorum through in vitro enzymatic assays. Similarly, Hsieh et al. [62,63] validated the function of green bamboo phenylalanine ammonia-lyase in E. coli and Pichia pastoris.

Due to the simplicity of *Arabidopsis* transformation, many bamboo gene functions were subsequently validated in this model plant. Examples include the roles of *PeMPEC* in photosynthesis and photoprotection [64], *NAC* in stress resistance [65], *PeUGE* in sugar transport [66], *ZEP* in photoprotection [67], *PeTIP4;1-1* in water transport and drought and salinity stress resistance [68], *PsbSs* in enhancing photoprotection and circumventing photoinhibition [69], and *PePIP2;7* in stress resistance [70]. However, *Arabidopsis* (a dicot) and bamboo (a monocot) exhibit significant phenotypic differences. For example, bamboo culms can reach heights of up to 20 m, whereas *Arabidopsis* stems are typically only about 1–3 mm in length. While *Arabidopsis* is suitable for studying stress resistance, photosynthesis, and energy transport, it is less effective for investigating bamboo growth, development, and wood formation.

Recognizing this limitation, researchers shifted to using rice, a closer monocot relative, for heterologous expression. Notable studies include the role of *PheLBD12* in regulating plant height [71], *PeDHN* in drought and saline-alkali tolerance [72], *PeHDZ23987* and *PeAAP29123* in nitrogen starvation tolerance [73]), *PheMTA1* and *PheMTA2* in root development and salt stress resistance [48], and *PeHDZ72* in drought tolerance by promoting sugar and water transport [74].

**Advances in bamboo transformation and gene editing.** In 2012, the genetic transformation of Ma bamboo was successfully achieved [14,15,75]. Ye et al. [13] further improved the transformation efficiency of Ma bamboo. Subsequently, Ye et al. [76] used the maize *UBI* promoter and rice *OsU6b* promoter to drive *Cas9* and sgRNA expression, enabling effective gene editing in Ma bamboo. Ma bamboo and green bamboo are sympodial bamboos, which are considered more amenable to explant dedifferentiation and redifferentiation than monopodial bamboos. Huang et al. [16] achieved genetic transformation and gene editing in Moso bamboo, a monopodial bamboo. However, the polyploid genomes (4x-6x) and high heterozygosity (0.06%~0.76% SNPs/kb) in woody bamboo may require inconsistent tissue culture conditions [77]. Despite these breakthroughs, reports on gene function validation in bamboo remain limited. For example, Zhang et al. [78] and Qiao et al. [15] used *Agrobacterium* EHA105 carrying the *CodA* gene under the *Rd29A* promoter to transform Ma bamboo anther-derived callus, obtaining transgenic plants with improved cold tolerance. Li et al. [75] transformed the 4-coumarate: CoA ligase gene (*4CL*) into Ma bamboo, producing transgenic plants. Ye et al. [13] and Xiang et al. [79] successfully transformed the maize *Lc* gene into Ma bamboo, resulting in anthocyanin accumulation. Wang et al. [80] demonstrated that the overexpression of ARF3 and ARF6 in the Ma bamboo background significantly altered lignin accumulation in transgenic bamboo plants. Lin et al. [81] edited the phytoene desaturase gene (*PDS*) in green bamboo, producing albino seedlings. Ye et al. [76] edited the *DlmGRG1* gene, resulting in plants with significantly increased internode length. Huang et al. [16] overexpressed the *GUS* gene and constructed a CRISPR/Cas9 vector using the Moso bamboo *U3* promoter to drive *PDS* sgRNA, obtaining *PDS*-mutated albino plants.

**Transient transformation and alternative methods.** In addition to stable transformation, transient transformation and gene editing systems have been developed for bamboo. Chen et al. [82] achieved the transient transformation of Moso and Ma bamboo using physical damage and vacuum infiltration-assisted *Agrobacterium* infection, successfully expressing the *GUS* gene in leaves and the *RUBY* gene in roots with 20–30% efficiency. Sun et al. [19] established an in planta transient expression system for exogenous genes in bamboo, successfully expressing the *RUBY* gene in leaf sheaths of Moso bamboo, *P. aureosulcata f. spectabilis* and *P. aureosulcata f. aureocarlis*. Although these methods do not yield stably inherited transgenic lines, they allow for the rapid acquisition of transgenic or gene-edited traits, making them suitable for preliminary gene function validation.

Furthermore, alternative approaches to *Agrobacterium*-mediated transformation have been explored. Hu et al. [83] used gene gun bombardment to transfer the *GUS* gene into mature Moso bamboo embryos. Yuan et al. [84] developed a PEG-mediated transformation method for Moso bamboo callus tissue. Lin et al. [81] developed a PEG-mediated gene transformation and editing system in green bamboo protoplasts. Chen et al. [82] achieved the PEG-mediated transformation of *GFP* into Moso and Ma bamboo protoplasts with efficiencies of 44.7% and 35.2%, respectively.

Zhang et al. [85] introduced multicolored fluorescent organelle marker genes into Moso bamboo protoplasts, developing a subcellular localization system. Jin et al. [86] developed a bamboo gene expression system using Bamboo mosaic virus (BaMV), successfully expressing exogenous genes (*EGFP* and *RUBY*) and endogenous genes (*ACE1* and *DEC1*) in Moso and Ma bamboo. Wu et al. [87] leveraged BaMV’s cargo capacity to transport Cas9 proteins, enabling targeted gene editing in non-infected bamboo tissues without integrating Cas9 fragments into the bamboo chromosome.

The development of transgenic overexpression and gene editing technologies has revolutionized bamboo genetic engineering. These advancements provide versatile tools for gene function analysis and pave the way for future precision breeding in bamboo research, contributing to our understanding of bamboo biology and its applications in sustainable agriculture and biotechnology.

## 3. Opportunities and Challenges in Bamboo Genomics

Bamboo, known for its rapid growth and excellent material properties, has a wide range of applications in daily necessities, building materials, and industrial products, including bamboo-based composites [88]. Although some bamboo products have been developed as alternatives to plastics, their adoption remains limited due to insufficient public awareness. The introduction of the “Bamboo as a Substitute for Plastic” initiative has provided strong policy support for the advancement of bamboo genomics. Increased attention from governments and society has also brought new research funding and collaborative opportunities to this field.

### 3.1. Policy Support, Social Impact, and Challenges

In 2022, the Chinese government and the International Bamboo and Rattan Organization (INBAR) jointly launched the “Bamboo as a Substitute for Plastic” initiative to combat plastic pollution, reduce carbon emissions, and promote sustainability. This initiative has garnered significant support from governments and society, creating both opportunities and challenges for bamboo genomics. For instance, on 24 June 2022, the initiative was included in the outcomes list of the High-Level Dialogue on Global Development. In October 2023, China’s National Development and Reform Commission (NDRC) and other departments issued the “Three-Year Action Plan for Accelerating the Development of Bamboo as a Substitute for Plastic,” setting the goal of establishing a preliminary industrial system for bamboo-based alternatives by 2025. On 7 November 2023, China and INBAR jointly released the Global Action Plan for Bamboo as a Substitute for Plastic. On 8 April 2024, the NDRC issued the “Special Management Measures for Central Budgetary Investment in Energy Conservation and Carbon Reduction,” which included bamboo-based product production as a key focus area for circular economy projects aimed at reducing carbon emissions.

In 2024, China’s National Natural Science Foundation announced a major research project titled “Structural Regulation and Directed Reorganization Mechanisms of Bamboo as a Substitute for Plastic.” This project focuses on key technologies such as bamboo structural regulation, fiber-directed reorganization, green separation, and controllable degradation, aiming to establish a new theoretical framework for bamboo-based materials. It seeks to provide scientific and technological support for replacing bulk plastic products in construction, packaging, and transportation, thereby addressing global plastic pollution and supporting China’s “Dual Carbon” strategy and the high-quality development of the Belt and Road Initiative (source: https://xww.csuft.edu.cn/ldxw/202502/t20250225_167642.html, accessed on 1 June 2025). These efforts highlight the substantial government support driving new opportunities in bamboo genomics.

### 3.2. Future Research Directions

Bamboo genomics stands at a pivotal juncture, driven by global policy support for sustainable development and the increasing demand for eco-friendly alternatives to plastic. While bamboo offers immense economic and ecological potential, its unique biological characteristics present both opportunities and challenges that must be addressed through advanced genetic research. This section outlines key future research directions aimed at unlocking bamboo’s full potential while addressing its inherent limitations.

**Expanding bamboo’s geographical range through genetic regulation.** Bamboo is an incredibly diverse plant, with over 1400 species distributed worldwide. Different bamboo species exhibit varying degrees of adaptability to environmental conditions [2], which contributes to their widespread natural distribution across tropical and subtropical regions. The natural distribution of bamboo is influenced by factors such as soil type, rainfall, temperature, and altitude. Bamboo can be found at latitudes ranging from 47° S to 50°30′ N and at altitudes from sea level up to 4300 m [1]. However, not all bamboo species can thrive in every specific environment, which poses challenges for their cultivation and utilization.

For the challenges in bamboo’s climatic adaptability, one of the most pressing challenges in bamboo cultivation is its climatic specificity. Bamboo primarily thrives in warm, lower altitudes, and humid environments, which restricts its geographical range [89]. For instance, Moso bamboo, a widely cultivated species, struggles to grow north of the Yellow River in China due to colder and drier conditions [90]. Additionally, light availability significantly affects bamboo’s photosynthetic rate [91] as well as the timing and quantity of new shoot growth [92]. This limitation hampers the widespread adoption of bamboo as a sustainable resource in diverse regions. On one hand, this restricts the cultivation range of high-quality bamboo species, limiting industrial-scale production. On the other hand, it increases transportation costs from production sites to markets.

In addition, the application of bamboo forests in soil conservation should not be overlooked. Bamboo, as a typical shallow-rooted, fast-growing clonal plant species, plays a crucial role in soil and water conservation due to its well-developed rhizome system and high canopy closure [93]. Therefore, expanding bamboo’s geographical range by cultivating drought-resistant, high-altitude-tolerant, and barren-soil-adapted bamboo species can significantly contribute to soil conservation, mitigating soil erosion, and preventing landslides.

For the potential of genetic regulation, plants’ ability to withstand environmental stress is regulated by their genes. To overcome this barrier, future research should focus on identifying and harnessing genetic factors associated with frost and drought resistance. By regulating the expression of key genes in bamboo, such as *CBL*, *TTG1*, *NPR3*, *PYL*, *NAC*, and *AP2/ERF* [94,95,96,97,98,99], researchers can introduce or enhance these traits in bamboo, enabling it to adapt to a broader range of climates. This genetic modification could not only expand bamboo’s cultivation range but also enhance its resilience to climate change, ensuring its viability as a sustainable resource in the face of global environmental shifts. By integrating environmental studies and genetic engineering, bamboo can not only adapt to a wider range of climates but also play a more significant role in the global sustainable resource market.

**Enhancing genetic transformation and gene editing efficiency.** Despite significant advancements, the efficiency of bamboo genetic transformation and gene editing remains suboptimal. Current methods often rely on heterologous expression in model organisms like rice and *Arabidopsis*, which, while useful, do not fully capture the complexities of bamboo biology. Additionally, many bamboo plants are polyploids with lengthy growth cycles—ranging from 3 to 150 years [10,11,12]. The existence of multiple gene copies complicates the generation of homozygous mutations in the T0 generation, posing a significant challenge to traditional breeding and genetic engineering efforts. To address these limitations, several strategies could be explored. Optimizing delivery methods, such as the use of nanoparticles [100], could enhance the precision and efficiency of gene delivery. Refining culture media and hormone regimes could help achieve the optimal callus state more consistently, thereby improving transformation efficiency. Additionally, seeking high-fidelity Cas variants and multiplex guide RNA designs [101] could improve gene-editing efficiency, enabling higher frequencies of homozygous or biallelic mutations. These advancements would not only address the current methodological constraints but also pave the way for more reliable and effective genetic modifications in bamboo.

To address these issues, future research should prioritize the development of more efficient *in planta* gene transformation methods, particularly for bamboo shoots and buds. This approach could significantly accelerate the acquisition of desirable traits, such as enhanced growth rates, disease resistance, and stress tolerance. Furthermore, unraveling the mechanisms of bamboo flowering could provide insights into reducing its growth cycle, making it more amenable to genetic improvement and biosafety considerations, such as the removal of exogenous genetic fragments through self-pollination.

**Tailoring bamboo for the “Bamboo as a Substitute for Plastic”.** The global “Bamboo as a Substitute for Plastic” initiative underscores the need to tailor bamboo’s properties for specific applications. We envision future bamboo-based products playing a significant role in construction, packaging, textile fibers for the garment industry, agricultural mulching films, and vehicle interiors. Each of these applications requires distinct bamboo characteristics to ensure optimal performance. For example, engineering or construction materials require bamboo with high strength, long internodes, thick culm wall, and long fibers [102]. In contrast, textile fibers for the garment industry materials require bamboo with an optimal length-to-diameter ratio, strength, cohesiveness, and torsional rigidity [103]. Packaging materials need fibers with a small microfibril angle and low crystallinity [7], while agricultural mulching films, on the other hand, require bamboo with long fibers and low lignin content [104]. By customizing bamboo to these specific requirements, the initiative can effectively address diverse industrial needs, offering sustainable and high-performance alternatives to plastic across multiple sectors.

While the complete replacement of plastic with bamboo may take several decades, our short-term goals should focus on understanding the molecular mechanisms underlying culm wall structure, fiber morphology, and molecular composition of bamboo. This foundational research will identify key genetic factors controlling these traits, providing a genetic resource pool for targeted improvement. By leveraging genetic engineering, researchers can tailor bamboo’s physical and chemical properties to optimize its functionality for specific applications. This targeted approach will not only enhance the plasticity and performance of bamboo products but also reduce energy consumption during their processing and production, further solidifying bamboo’s role as a sustainable alternative to plastic.

The future of bamboo genomics is brimming with potential, but it also faces significant challenges that must be addressed through targeted research and innovation. These can be addressed by expanding bamboo’s geographical range, enhancing genetic transformation efficiency, and tailoring its properties for specific applications. These efforts are critical in ensuring that bamboo remains at the forefront of sustainable development, offering a viable alternative to plastic and contributing to a greener, more sustainable future. If these challenges are not addressed, bamboo’s pursuit of sustainable alternatives may be overshadowed by other species, such as *Calotropis gigantea* [105], *Zea mays* [106], and seaweed [107]. However, with a focused research agenda and the application of advanced genetic technologies, bamboo can solidify its position as a cornerstone of the global sustainability movement.

## 4. Conclusions

Bamboo genomics stands at a pivotal juncture, driven by the urgent need for sustainable alternatives to plastics and the growing recognition of bamboo’s ecological and economic potential. The “Bamboo as a Substitute for Plastic” initiative, supported by robust policy frameworks and international collaboration, has provided a significant impetus for advancing research in this field. However, the realization of bamboo’s full potential as a sustainable material hinges on overcoming several scientific and technical challenges. Key areas of focus include enhancing the efficiency of genetic transformation and gene editing technologies, expanding bamboo’s cultivation range through the identification of stress-resistant genetic factors, and developing innovative methods such as *in planta* gene transformation [19] to accelerate trait acquisition. Additionally, unraveling the mechanisms of bamboo flowering and addressing biosafety concerns are critical to ensuring the long-term success of genetic improvement efforts. The integration of multidisciplinary or transdisciplinary approaches, from molecular biology to material science, will be essential to establish bamboo as a viable and scalable alternative to plastics. By addressing these challenges, bamboo genomics can not only contribute to reducing global plastic pollution but also support broader sustainability goals, including carbon reduction and the promotion of circular economies.

In summary, although the path forward is complex, the alignment of policy support, scientific innovation, and international collaboration presents a unique opportunity to leverage bamboo as a cornerstone of sustainable development. Future research must focus on bridging the gap between scientific discovery and practical application, ensuring that bamboo can fulfill its promise as a transformative resource for a greener future.

## Data Availability

The data that support the findings of this study are available from the corresponding authors upon request.
